# Perspectives on the Development of Immune Memory Associated with Vaccination

**DOI:** 10.3390/vaccines14050420

**Published:** 2026-05-07

**Authors:** Pu He, Yuhe Zhou, Yu Feng, Ting Zhou, Fei Li, Youjun Mi, Wenhua Zhang, Bingdong Zhu

**Affiliations:** 1 School of Life Science, Lanzhou University, Lanzhou 730000, China; 18993596041@163.com; 2 State Key Laboratory of Animal Disease Control and Prevention and Lanzhou Center for Tuberculosis Research, Institute of Pathogen Biology, School of Basic Medical Sciences, Lanzhou University, Lanzhou 730000, China; zhouyuhe2025@lzu.edu.cn (Y.Z.); fyu2024@lzu.edu.cn (Y.F.); zhouting2025@lzu.edu.cn (T.Z.); lifei2013666@163.com (F.L.); miyoujun@126.com (Y.M.); 3 College of Veterinary Medicine, Lanzhou University, Lanzhou Veterinary Research Institute, Chinese Academy of Agricultural Sciences, Lanzhou 730000, China

**Keywords:** immune memory, vaccine, metabolic reprogramming, epigenetic modifications, immune aging

## Abstract

The goal of vaccination is to induce long-term immune memory. Traditionally, immune memory has been thought to be mediated by memory T cells and B cells. In recent years, trained immunity mediated by the innate immune cells (e.g., NK cells, neutrophils, and monocytes/macrophages) has garnered increasing attention. Trained immunity exhibits an antigen-nonspecific immune memory that provides broader protection against heterologous infections. This article reviews the mechanisms involved in the development of trained immunity, memory T cells, and B cells with a particular focus on metabolic reprogramming and epigenetic modifications. Moreover, the effects of aging on immune memory and the factors involved in regulating the vaccine-induced immune memory in older people are discussed. By understanding immune memory and its regulatory mechanisms, we can better design vaccines and optimize vaccination strategies to induce long-lasting immune memory.

## 1. Introduction

Protective immunity is mediated by both innate and adaptive immune responses. Innate immunity, the first line of host defense, detects pathogens and tissue damage signals and responds by initiating a range of molecular and cellular processes that eliminate pathogens within a short time (several hours to days) and limit tissue damage. The innate immune system is primarily mediated by phagocytic cells, including macrophages and dendritic cells. Studies have shown that innate immune cells can also develop a form of antigen-nonspecific memory termed ‘trained immunity’, which is characterized by enhanced responsiveness to subsequent heterologous infections through epigenetic and metabolic reprogramming [1]. Adaptive immunity provides a higher degree of antigen specificity through the expression of antigen-specific T-cell receptors (TCRs) and B-cell receptors (BCRs) on lymphocytes. Upon antigen encounter, lymphocytes are activated and differentiate into effector cells to address immediate threats, while also generating memory cells that protect against potential reinfections. Memory cells include memory T cells, memory B cells, and long-lived plasma cells (LLPCs). These cells primarily reside in lymph nodes, the spleen, tissues, and some also circulate in the bloodstream. Antibodies, produced by memory B cells and plasma cells, serve as key soluble mediators against pathogens present predominantly in the blood and mucosal secretions.

Vaccination has been a cornerstone of infectious disease prevention since the introduction of the smallpox vaccine. The smallpox vaccine achieved remarkable success in controlling and ultimately eradicating this devastating global disease, representing one of the greatest public health achievements in human history. However, developing effective vaccines against chronic intracellular pathogens, which need the involvement of memory T cells, remains a significant challenge. For many years, vaccine development relied largely on empirical approaches rather than the systematic application of immunological principles [2]. With the rapid advances in immunology over recent decades, researchers have increasingly applied fundamental immunological concepts to elucidate the mechanisms underlying vaccine-induced protection and to design novel vaccines for both the prevention of infectious diseases and the treatment of cancer. Research indicates that trained immunity contributes to the non-specific protective effects of certain vaccines (e.g., BCG), providing insights into strategies for enhancing vaccine efficacy beyond traditional adaptive immune memory. In this review, we systematically summarize the characteristics of vaccine-induced immune memory and the key cellular and molecular mechanisms involved. We also explore the multidimensional factors that influence the establishment and maintenance of immune memory (including both adaptive memory and trained immunity) and discuss potential strategies to enhance vaccine efficacy through the modulation of immune memory pathways.

## 2. Materials and Methods

We conducted a narrative review of the literature on the immune memory, with a focus on studies examining vaccine-induced immune memory and the key cellular and molecular regulatory mechanisms involved. A comprehensive literature search was conducted across multiple electronic databases, such as PubMed, Scopus, Web of Science, ScienceDirect, and Google Scholar, to identify relevant publications available by January 2026. The search was restricted to articles published in English. Conference abstracts lacking full-text content, editorial letters, and non-peer-reviewed materials were generally excluded, unless they provided critical conceptual insights. The search strategy combined terms related to how immune memory is regulated by epigenetics and metabolic reprogramming, including the effects of immune aging, such as “immune memory”, “T cell”, “B cell”, “trained immunity”, “epigenetic regulation”, “DNA methylation”, “histone modification”, “chromatin remodeling”, “metabolic reprogramming”, “immune aging”, and “Vaccine efficacy”. Boolean operators (AND, OR) were used to refine the search, for example (“epigenetic regulation” OR “DNA methylation” OR “histone modification” OR “chromatin remodeling”) AND (“immune memory” OR “trained immunity”) AND (“T cells” OR “B cells”). Studies were selected based on their alignment with the review’s scope, with priority given to publications offering mechanistic insights into (i) epigenetic regulation of immune memory, (ii) metabolic reprogramming and its role in orchestrating immune cell fate and memory, and (iii) the impact of aging on immune memory. Given the heterogeneity of the available evidence and the exploratory nature of this field, a strictly formal systematic review framework was not applied. Instead, a qualitative synthesis approach was adopted to integrate the findings, with the aim of constructing a comprehensive overview of current understanding, identifying promising new hypotheses, and highlighting critical knowledge gaps. This narrative method, which is inherently more susceptible to selection bias than a systematic review, was chosen to facilitate the integration of mechanisms from a diverse and rapidly evolving field of research.

## 3. Principles of Immune Memory and Memory Cells

**Figure 1:**
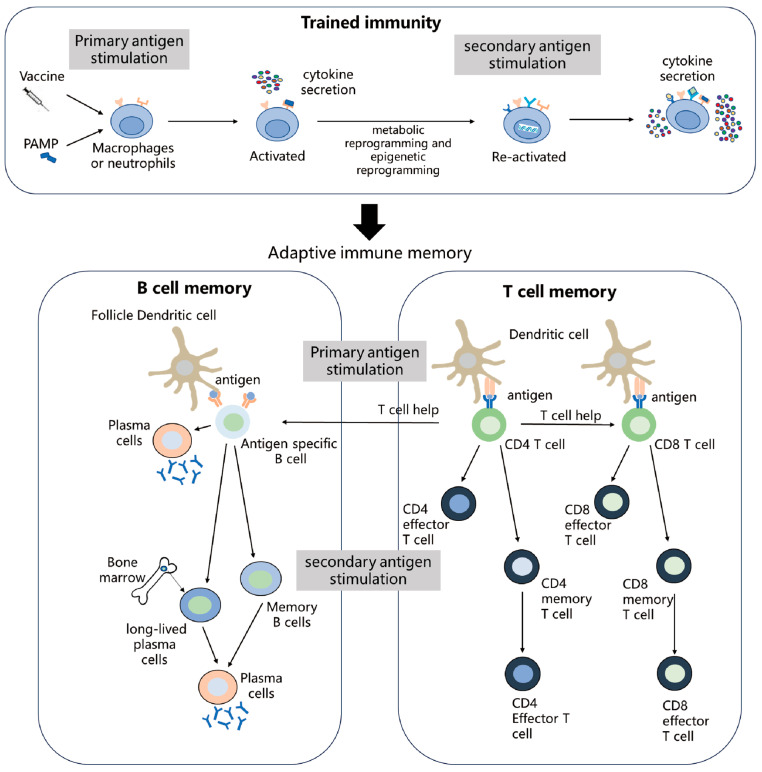
Vaccine-induced immune memory formation. Macrophages or neutrophils are activated through Toll-like receptor (TLR) signaling. Upon infection, these innate immune cells exhibit an antigen-nonspecific response, characterized by increased production of pro-inflammatory cytokines. In addition, dendritic cells present antigen to naïve T cells, and initiate the development of antigen-specific memory CD4^+^ T cells, CD8^+^ T cells, B cells, and LLPCs. Upon secondary stimulation, memory B and T cells respond more rapidly, differentiating into effector T cells and plasma cells, which provide enhanced immune protection. LLPCs secrete antibodies.

Immune memory is a hallmark of adaptive immunity, characterized by its ability to persist long after antigen clearance [3,4], thereby driving a sustained and enhanced secondary response upon subsequent encounters with the same antigen (Figure 1). Adaptive immune memory cells can persist for extended periods in the circulation and in specific tissues, even in the absence of an antigen. Distinct memory subsets differ significantly in their capacity for self-renewal, lifespan, and effector function [5]. In contrast, trained immunity manifests as an antigen-nonspecific, broader secondary enhanced immune response. The duration of trained immunity is also shorter compared to adaptive immune memory (Box 1).

Box 1 **Trained immunity and adaptive immune memory**

Trained immunity refers to the ability of hematopoietic stem and progenitor cells and innate immune cells to acquire adaptive-like characteristics, leading to long-term functional changes. Trained immunity is antigen-nonspecific and it provides broader protection against heterologous infections. The duration of trained immunity effects, as demonstrated in immunological studies, can be maintained for weeks to months or up to a couple of years. For example, BCG vaccination can induce trained immunity, offering protection against lethal *Candida albicans* infection [6].

Adaptive immune memory is characterized by antigen specificity and clonal expansion, enabling a protective immune response that persists for years to decades after antigen clearance. This form of memory, mediated by T cells, B cells (including their plasma cell progeny), and their secreted antibodies, is essential for human survival against recurrent infections. For example, the smallpox vaccine can confer lifelong protection following vaccination, with antigen-specific IgG antibodies persisting for decades [7].

### 3.1. Trained Immunity

The molecular mechanisms that facilitate the induction of trained immunity are likely represented by an intersection of immunologic signaling pathways, alterations in cellular metabolism, and long-term epigenetic modifications. Activation of pattern recognition receptors on the membrane of innate immune cells stimulates diverse intracellular pathways, with activation of transcription factors for the induction of the transcription of genes necessary for antimicrobial host defense, such as proinflammatory cytokines, chemokines, and defensins [8]. The induction of trained immunity activates various metabolic pathways that serve, on one hand, as a source of nutrients and, on the other hand, as a source of metabolites that drive epigenetic modifications and the remodeling of chromatin architecture. Gene transcription is linked to epigenetic changes that enhance chromatin accessibility, including histone methylation and acetylation. Some of these epigenetic modifications persist even after the stimulus is removed, facilitating a faster and more efficient response upon subsequent re-stimulation of the cells [1,9]. In humans, trained immunity is considered one of the mechanisms by which live attenuated vaccines, such as BCG, MMR (the Measles, Mumps, and Rubella Vaccine), and OPV (the oral poliovirus vaccine), confer heterologous protection against unrelated infections [10]. Overall, trained immunity represents a long-term functional reprogramming of innate immune cells driven by metabolic–epigenetic coupling.

### 3.2. Memory T Cells

Memory T cells are characterized by their rapid recall response. Memory T cells are commonly identified based on their capacity to produce effector cytokines and the expression of the adhesion molecule CD44 in mice [11]. Memory T cells are primarily classified into central memory T cell (T_CM_), effector memory T cell (T_EM_), and tissue-resident memory T cell (T_RM_) subsets, although additional populations such as T memory stem cells (T_SCM_) have also been described [12]. T_CM_ cells are relatively quiescent and are characterized by the low expression of effector molecules and high expression of lymphoid tissue homing receptors, such as CCR7 and CD62L. They predominantly produce IL-2 rather than IFN-γ [13,14]. In contrast, T_EM_ cells have a shorter lifespan, express low levels of lymphoid homing markers, and circulate between the bloodstream and inflamed non-lymphoid tissues; they primarily secrete IFN-γ [14]. T_RM_ cells permanently reside in peripheral tissues, are characterized by the expression of CD103 and CD69, and function as a first line of defense against reinfection [15,16].

The formation of T-cell memory involves three pivotal stages: the transition from effector to memory, homeostatic maintenance, and tissue residency. The transition from effector to memory is regulated by the strength of TCR stimulus. Strong signals drive forming effector cells and weaker signals promoting memory cell fate and self-renewal manifested by the maintenance of TCF1 expression [17,18]. The AKT-mTORC1 axis plays a key role in fate decision, which activates glycolysis pathway in effector T cells and endows them with faster proliferation activity [19]. Cytokines are critical molecules that regulate the maintenance of memory T-cell homeostasis and long-term survival. IL-7 and IL-15 are important for the development of T cells, and promote the survival and homeostasis proliferation of T cells [20]. The transcription factors HOBIT, BLIMP1, RUNX3 and NOTCH have all been implicated in driving T_RM_ cell generation, tissue residency and maintenance in mice [21,22,23].

### 3.3. Memory B Cells and LLPCs

Long-term humoral immunity relies on a coordinated “long-lived plasma cell and memory B cell” system [24]. Upon primary exposure to a pathogen, antigens drive the generation of LLPCs and memory B cells within secondary lymphoid organs, particularly in the germinal center (GC) [25,26,27]. This process can be broadly divided into two stages. In the first stage, antigen engagement of the BCR activates naïve B cells, promoting their differentiation into short-lived plasma cells and GC B cells within lymphoid follicles. In the second stage, under sustained antigenic stimulation and appropriate helper signals, GC B cells further differentiate into LLPCs and memory B cells, thereby establishing the foundation of long-term humoral immunity. Upon re-exposure to the pathogen, pre-existing LLPCs residing in specialized survival niches, such as the bone marrow, rapidly secrete antibodies to maintain circulating antibody levels, constituting the first line of adaptive immune defense. Subsequently, memory B cells are activated and undergo rapid clonal expansion, generating highly specific protective responses against both the original pathogen and its variants [24].

The establishment and maintenance of B-cell immune memory are influenced by multiple factors, including the duration and nature of antigen persistence, the magnitude and dynamics of GC reactions, the quality of follicular helper T cells, and antigen stability and diversity. Various activation conditions regulate different fates for B cells; for instance, strong antigen reactivity and high antigen dose enhance the formation of ASCs [28,29]. By contrast, B cells with weak affinity for antigens that receive weak signals from TFH cells become GC-independent MBCs [30]. A crucial transcription factor that influences the differentiation fate of activated B cells is IRF4. Strong BCR signaling sustains IRF4 expression, while weaker BCR signaling downregulates IRF4 and facilitates BCL6-mediated differentiation into germinal center B cells [31,32]. The antigen affinity of individual GC B cells dictates their fate by controlling how well they can compete for signals from GC TFH cells. The fate and lifespan of plasma cells depend on their tissue localization. LLPCs were originally discovered in the bone marrow and have a lifespan of 140 days, compared to just 3 days for plasma cells in the spleen [33,34].

### 3.4. The Regulation of Immune Memory

Immunological memory is tightly regulated through the coordinated interplay of antigen characteristics, cytokine networks, and transcriptional programs. Features of antigen exposure, such as persistence, dose, and immunogenicity, directly influence the strength and duration of T- and B-cell activation, thereby shaping the magnitude and diversity of memory cell populations. Cytokines play essential roles in this process. IL-2 [35], IL-7 [36,37] and IL-15 [38] are critical for the survival and homeostatic proliferation of memory T cells. At the transcriptional level, key regulators including Id-3 [39], Bcl-6 [40], TCF1 [41] and FOXO1 [42] orchestrate lineage commitment and functional plasticity by controlling gene expression programs associated with memory cell differentiation, maintenance, and recall capacity. Collectively, these components form an integrated regulatory network that enables the immune system to mount rapid, robust, and durable protective responses upon re-encounter with pathogens. Besides these, recent studies show that immune memory is regulated by epigenetics [43] and metabolic reprogramming [44]. In addition, in vaccination for older people it is usually difficult to generate effective immune memory [45]. These problems are discussed in detail in the review.

## 4. Epigenetics Regulates Immune Memory

**Figure 2:**
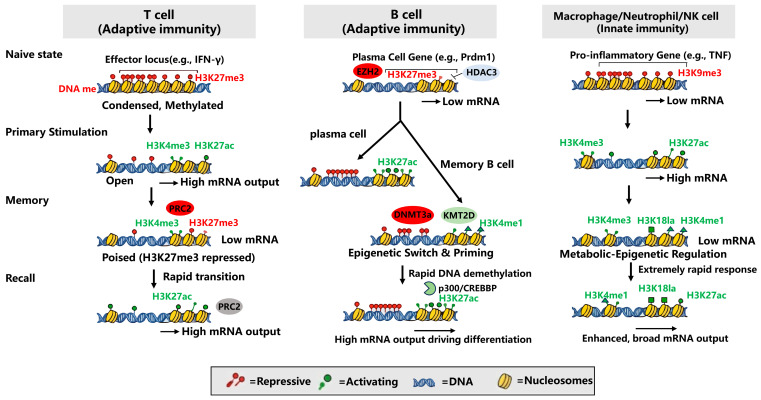
DNA methylation and histone modification in the formation of immune memory. For T cells, effector loci (e.g., IFN-γ) are heavily methylated and condensed at naive state. Loci adopt a “poised” configuration at the memory state. Chromatin remains accessible (retaining H3K4me3) but is temporarily silenced by PRC2-mediated H3K27me3 to prevent exhaustion. Rapid removal of H3K27me3 permits H3K27ac re-acquisition and robust transcription at the recall response. For B cells, memory commitment requires stable silencing of plasma cell genes (Prdm1) via DNMT3a-mediated de novo DNA methylation, alongside KMT2D-driven enhancer priming (H3K4me1). Secondary differentiation is driven by rapid DNA demethylation and p300/CREBBP-mediated H3K27ac deposition at the recall response. For trained immunity, initial stimulation (e.g., BCG) induces chromatin opening and H3K4me3/H3K4me1 deposition at pro-inflammatory loci (e.g., TNF) in the naive state. Chromatin accessibility is maintained. Metabolic reprogramming is stably recorded via histone lactylation (H3K18la) at the memory state. Pre-primed loci undergo extremely rapid H3K27ac enrichment for an enhanced, broad transcriptional output at the recall response.

Through regulatory mechanisms such as DNA methylation, histone modifications, and non-coding RNAs (ncRNAs), memory cells can stably maintain their immune-associated phenotypes and rapidly initiate effector programs upon secondary stimulation. Specifically, the core commonalities among memory T cells, memory B cells, and innate immune cells at the level of DNA methylation and histone modifications are primarily focused on the following: In the naive state, cellular chromatin is highly condensed, and effector genes are repressed to maintain immune homeostasis. Primary stimulation induces chromatin opening, DNA demethylation, and the enrichment of activating epigenetic marks. During the memory maintenance phase, effector genes establish a “poised” configuration—retaining H3K4me3/H3K27ac marks to preserve chromatin accessibility, while relying on H3K27me3 or DNA methylation to enforce transcriptional quiescence, thereby pre-venting cellular exhaustion or premature terminal differentiation. Upon secondary stimulation, this pre-established epigenetic landscape enables cells to instantaneously relieve repression and recruit transcriptional complexes, rapidly mounting a robust secondary immune response (Figure 2). A deeper understanding of these regulatory mechanisms not only clarifies the molecular basis of immune memory but also provides a theoretical foundation for optimizing vaccine design and developing targeted interventions for immune-related diseases.

### 4.1. Epigenetic Basis of Trained Immunity

In contrast to adaptive immune memory, trained immunity depends on antigen receptor rearrangement; the establishment, maintenance, and functional execution of trained immunity are governed primarily by epigenetic regulation. Core mechanisms including chromatin remodeling, histone modifications, DNA methylation, and non-coding RNA-mediated regulation act in concert to form the central regulatory network underlying trained immunity.

#### 4.1.1. Heritable Remodeling of Chromatin State

The longevity and rapid responsiveness of trained immunity are rooted in stable alterations of chromatin architecture that establish a distinct “primed” cellular phenotype. This state is characterized by three core features: ① The opening of 3D chromatin architecture occurs as Topologically Associating Domains (TADs) facilitates stable three-dimensional looping contacts that precisely align immune genes with their regulatory elements, thereby reducing transcriptional stochasticity. Following vaccine-induced exposure in murine in vivo vaccine models (utilizing BCG immunization), ATAC-seq analyses have shown that hematopoietic stem cells undergo early chromatin accessibility remodeling following BCG exposure. Importantly, portions of this accessible chromatin landscape are retained after differentiation into myeloid or lymphoid lineages, providing an epigenetic basis for trained immunity in progeny cells [46]. ② Enrichment of activating epigenetic marks. Key pro-inflammatory genes and pattern recognition receptors (PRRs) acquire activating histone marks. H3K4me3 remains enriched at promoters of inflammatory genes such as TNF, IL-6, and IL-1β [47,48]. Distal enhancer regions display coordinated enrichment of H3K4me1 and H3K27ac [1]. For example, increased H3K27ac at the upstream regulatory region of NOD2 is a critical molecular event in BCG-induced trained immunity [49]. ③ Repressive epigenetic marks are selectively reduced at pro-inflammatory loci. Levels of H3K9me3 and H3K27me3, associated with heterochromatin formation and transcriptional repression, decline at inflammatory gene regions, thereby relieving transcriptional inhibition. In contrast, promoters of anti-inflammatory genes (e.g., IL-10) remain epigenetically silenced, preventing excessive immunosuppression [50]. Together, these coordinated chromatin modifications establish a durable yet flexible epigenetic framework that underlies the enhanced responsiveness characteristic of trained immunity.

#### 4.1.2. Multidimensional Epigenetic Regulatory Mechanisms Synergistically Drive the Establishment and Maintenance of Trained Immunity

The epigenetic regulation of trained immunity is not driven by a single mechanism but rather by an integrated network involving histone modifications, DNA methylation, ncRNAs, and 3D chromatin architecture. These regulatory layers function cooperatively to ensure precise control of gene expression. Among them, histone modifications play a central role in remodeling chromatin states and work in concert with 3D chromatin organization to activate trained immunity programs. A hallmark of trained immunity-associated gene activation is the “dual-mark” pattern consisting of H3K4me3 at promoters and H3K27ac at enhancers. For example, β-glucan stimulation increases H3K4me1 deposition at enhancer regions of the IL-32 gene in human monocytes [51]. Furthermore, β-glucan promotes the formation of H3K27ac modifications at enhancer regions in human monocytes. This mechanism partially reverses the state of tolerance induced by lipopolysaccharide (LPS) in macrophages in vitro, thereby facilitating the transcriptional reactivation of non-responsive genes [52]. Similarly, BCG exposure enhances H3K4me3 enrichment at effector gene loci in neutrophils, thereby potentiating cytokine production and antimicrobial activity [53]. The Mixed-Lineage Leukemia (MLL) complex mediates H3K4me3 deposition at target gene promoters [54]. Meanwhile the KDM4 family of histone demethylases serves as an important regulator of trained immunity. Inhibition of KDM4 significantly weakens β-glucan or BCG-induced training effects and reduces pro-inflammatory gene expression [55]. Recent studies have showed the BCG can induce the histone lactylation (H3K18la) as a novel regulatory mechanism. Lactate generated during metabolic reprogramming induces H3K18la, converting transient metabolic signals into more stable epigenetic marks and sustaining heightened innate immune responsiveness [56].

DNA methylation regulates the methylation status of gene promoters, thereby coordinating the selective activation or silencing of genes associated with trained immunity. It also plays a key role in shaping inter-individual variability in trained immune responses. Following BCG vaccination, promoter regions of immune-related genes, such as IL-1β, exhibit significant DNA demethylation. This loss of methylation not only serves as a molecular hallmark of trained immunity activation but also has potential as a predictive biomarker of individual responsiveness to training stimuli [57].

NcRNAs fine-tune trained immunity by acting as molecular guides that recruit epigenetic modification complexes to specific genomic loci. In this way, they function as key mediators linking chromatin architecture with epigenetic regulation. Immune-priming lncRNAs (IPLs) utilize pre-existing chromosomal loops to recruit H3K4me3-modifying complexes to promoters of innate immune genes. For example, IPL-IL1 mediates BCG-induced training in neutrophils [53]. Notably, insertion of an IPL within a chemokine-associated TAD can confer β-glucan-induced trained immunity to genes that were previously unresponsive. Similarly, UMLILO, an lncRNA located upstream of an inflammatory chemokine gene cluster, binds WD repeat domain 5 (WDR5) to recruit the MLL complex, promoting H3K4me3 deposition at promoters of chemokines such as CXCL1, CXCL2, CXCL3, and IL-8. Inhibition of UMLILO abolishes β-glucan-induced trained immunity [54]. MicroRNAs also contribute to this regulation. miR-155 enhances adaptive-like memory formation in NK cells during murine cytomegalovirus (MCMV) infection by targeting negative regulators of trained immunity, including NOXA, SOCS1, and SHIP1 [58]. At the same time, miR-155 upregulation is associated with myeloid-cell hyperactivation, highlighting its dual role in calibrating the magnitude of trained immune responses.

### 4.2. Epigenetic Remodeling the Formation of Memory T Cells

#### 4.2.1. Histone Modifications Modulating Chromatin Accessibility

Histone modifications represent one of the most active and dynamic aspects of epigenetic regulation in T cells [59]. A feature of memory T cells is the pre-establishment of activating histone marks at the promoter and enhancer regions of effector genes. The most well-characterized examples are H3K4me3 and H3K27ac. H3K4me3 is a stable marker of transcriptional activation, and in memory T cells it remains enriched at effector gene loci-such as IFN-γ even when the cells are in a resting state [60]. This relatively “open” chromatin configuration facilitates rapid recruitment of RNA polymerase, enabling swift transcriptional activation upon secondary stimulation. Similarly, in murine memory CD8^+^ T cells, H3K27ac contributes to the establishment of the memory transcriptional program and the capacity for rapid recall responses by marking active enhancers and maintaining an open chromatin state [61]. In contrast, H3K27me3 is a well-established repressive mark catalyzed by the Polycomb Repressive Complex 2 (PRC2). In memory T cells, H3K27me3 is deposited at genes associated with early effector differentiation, thereby preventing terminal differentiation or functional exhaustion.

#### 4.2.2. DNA Methylation Affecting Gene Transcription

DNA methylation, adding a methyl group (5-mC) to cytosine residues, generally suppresses gene transcription. Studies have demonstrated that DNA methylation plays a critical role in T-cell activation, differentiation, and the establishment of immune memory [62]. DNMT3a, a key DNA methyltransferase, regulates the expression of genes associated with survival and effector functions of T cells. T cells lacking DNMT3a are more likely to differentiate into long-lived memory T cells, ultimately enhancing immune memory and secondary immune responses [63]. In murine memory T cells, gene loci encoding cytotoxic molecules and pro-inflammatory cytokines—such as perforin, Granzyme B (Gzmb), and IFN-γ—are typically maintained in a highly methylated or re-methylated state [64]. This methylation pattern suppresses gene expression during the resting phase of memory T cells, promoting long-term survival. However, compared with naïve T cells, these loci exhibit relatively lower methylation levels, reflecting a “primed” epigenetic state that enables rapid de-repression and expression upon secondary stimulation, thereby ensuring swift effector responses [65]. Genes encoding key proteins involved in memory T cells maintenance and survival, such as IL-7Rα (CD127) and TCF7 (TCF-1), maintain a stable hypomethylated status in memory T cells [66]. This persistent demethylation provides a foundation for rapid transcriptional activation and sustained expression [67].

#### 4.2.3. NcRNAs Regulating the Expression of Important Molecules

NcRNAs play critical regulatory roles in T-cell function. MicroRNAs, in particular, influence the development, differentiation, and maintenance of memory T cells, including CD8^+^ T_CM_, and contribute to fate determination. For example, miR-181a promotes T_CM_ development by modulating TOX expression, whereas miR-155 supports regulatory T-cell (Treg) development by influencing the maturation of medullary thymic epithelial cells. These miRNAs are essential for maintaining immune homeostasis and sustaining immune memory [68]. In addition, emerging evidence suggests that long non-coding RNAs (lncRNAs) also participate in regulating T-cell development and function. For instance, the lncRNA Snhg1 modulates the Vps13D-dependent vesicular transport pathway, thereby ensuring proper expression and function of IL-7R in murine memory T cells. By enhancing IL-7 signaling, Snhg1 promotes the survival and functional maintenance of memory T cells [69].

### 4.3. Epigenetic Mechanisms Underlying Memory B-Cell Formation

The generation of memory B cells is orchestrated through extensive epigenetic reprogramming. During rapid proliferation and affinity maturation, GC B cells undergo dynamic DNA methylation, histone modifications, and chromatin remodeling, ultimately establishing a “poised” chromatin state characterized by the coexistence of activating and repressive marks [70]. This distinctive epigenetic configuration enables memory B cells to remain transcriptionally quiescent during the resting phase while retaining the capacity for rapid de-repression and activation of proliferation and differentiation programs upon antigen re-exposure. Such reprogramming provides a critical molecular basis for the efficiency and durability of vaccine-induced immunological memory.

#### 4.3.1. The Role of DNA Methylation

DNA methylation represents a regulatory layer that determines whether GC B cells exit the proliferative program and commit to a memory fate. GC B cells generally display a relatively hypomethylated genome, within which DNMT1 is essential for GC establishment and maintenance [71]. As differentiation proceeds toward memory B cells or plasma cells, the DNA methylation landscape undergoes extensive remodeling. Epigenomic analyses have shown that MBCs exhibit increased DNMT3a expression and reduced DNMT3b expression compared with GC B cells. This “de novo methyltransferase switch” is thought to promote the silencing of plasma cell differentiation genes (e.g., *Prdm1*) while stabilizing memory-associated transcriptional programs [72]. Concurrently, TET family-mediated DNA demethylation enables the expression and functional activity of activation-induced cytidine deaminase (AID), ensuring that commitment to the memory fate occurs only after affinity maturation is complete [73]. Furthermore, the HELLS-DNMT1 axis reinforces temporal regulation of this methylation transition by maintaining the GC phenotype and delaying premature MBC selection [74].

#### 4.3.2. Histone Methylation Orchestrating the Differentiation of GC B Cells into MBCs

At the level of histone modification, the differentiation of GC B cells into MBCs is regulated by a dynamic balance between repressive and activating methylation marks. As a core component of the PRC2, EZH2 catalyzes the deposition of the repressive mark H3K27me3, thereby silencing differentiation-associated genes, including *Prdm1* (encoding BLIMP1) and *Irf4*, during the GC stage. This repressive activity maintains the highly proliferative state of GC B cells and prevents their premature differentiation into memory or plasma cell fates [75,76]. Accordingly, loss of EZH2 markedly impairs the GC reaction and reduces the generation of functional MBCs [75,77]. In contrast, KMT2D mediates H3K4me1 at enhancer regions, establishing a transcriptionally permissive landscape that supports the transition of GC B cells toward the MBC lineage by activating genes involved in cell survival and cell cycle regulation [78].

#### 4.3.3. Histone Acetylation and Deacetylation Locking the Quiescent Memory State and Inhibiting Plasma Cell Differentiation

Histone acetylation represents a finely tuned regulatory mechanism in the formation and maintenance of MBCs. In GC B cells, the BCL6-SMRT complex recruits HDAC3 to deacetylate and silence enhancers associated with plasma cell differentiation, particularly Prdm1, thereby preserving the GC or memory-precursor state [79]. In contrast, investigations using human ex vivo and in vitro models (specifically, primary tonsillar GC B cells and B-cell lymphoma lines) reveal that under steady-state conditions, acetylation mediated by p300/CREBBP can reactivate these enhancers once BCL6 repression is lifted, thereby promoting progression toward plasma cell differentiation [80]. MOZ further supports the non-plasma cell fate during MBC development by enhancing H3K9ac; its deficiency results in the generation of immature and functionally impaired MBC populations [81]. Collectively, the acetylation “switch” regulated by the dynamic interplay between HDAC3 and p300/CREBBP/MOZ ensures that MBCs remain in a differentiation-repressed state during quiescence while retaining the capacity for rapid reactivation and robust secondary responses.

#### 4.3.4. NcRNAs and Chromatin Topology

Beyond canonical epigenetic modifications, ncRNAs and higher-order chromatin architecture collectively contribute to the long-term stability of MBC fate. MicroRNAs, including miR-155, miR-125b, and miR-15a/16, fine-tune the magnitude of the GC reaction, memory formation, and cell survival by targeting regulators such as AID, Blimp-1, and Bcl-2 [82,83,84]. In addition, memory B cells progressively acquire IRF4-dependent changes in chromatin accessibility during primary immunization. Upon antigen re-exposure, these epigenetic imprints influence the reciprocal regulation of Blimp-1 and Bach2, thereby determining whether memory B cells preferentially re-enter secondary GCs or differentiate into plasma cells [85]. In murine models, Chromatin re-modelers such as ARID1A cooperate with transcription factors PU.1 and NF-κB to promote the expression of memory-associated genes (e.g., *Bach2* and *Hhex*), further stabilizing the MBC phenotype [86]. This process, often described as the “epigenetic recording of antigenic history”, provides a molecular framework for understanding the quality, durability, and functional adaptability of vaccine-induced immunological memory.

## 5. Metabolic Reprogramming Orchestrates Immune Cell Fate and Memory

**Figure 3:**
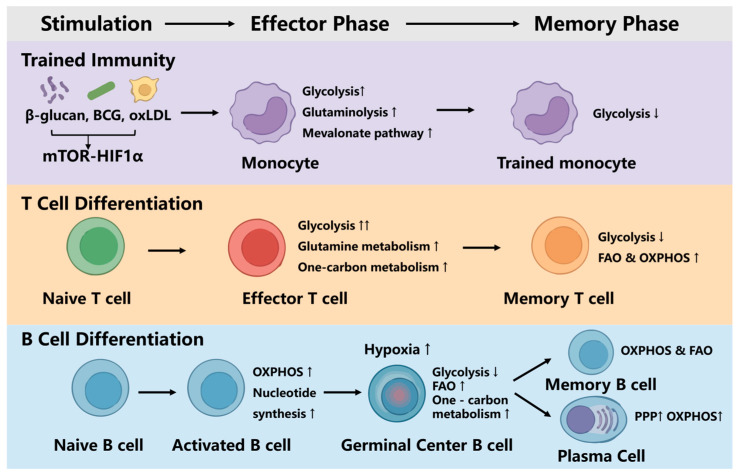
Metabolic responses characterize the formation and maintenance of immune memory. Upon stimulation, trained innate cells and effector T cells undergo glycolysis-dominant metabolic reprogramming, generating metabolites that promote epigenetic remodeling and imprinting. During memory differentiation, T cells shift toward FAO/OXPHOS-supported oxidative metabolism, whereas germinal center B cells preferentially engage FAO and one-carbon pathways under hypoxia, and plasma cells adopt glucose- and pentose phosphate pathway (PPP)-driven anabolic programs. “↑” indicates enhanced response or elevated expression level, “↓” represents reduced response or decreased expression level. “↑↑” indicates a more significant enhancement of the reaction.

Immune response is intrinsically coupled to metabolic reprogramming. Beyond merely sustaining bioenergetics and biosynthesis, metabolic networks generate rate-limiting signaling intermediates—including acetyl-CoA, lactate, fumarate, etc.—that orchestrate epigenetic remodeling and dictate gene expression [87,88]. The establishment of adaptive immune memory and trained immunity are all highly coordinated processes that integrate host metabolic remodeling, epigenetic regulation, and microbial-derived signals. Importantly, metabolic–epigenetic coupling acts as a central organizing principle; it translates environmental nutrient availability and antigenic stimuli into durable chromatin remodeling, thereby determining the longevity and functionality of memory subsets (Figure 3).

### 5.1. Metabolic Regulation of Innate Immune Memory

The induction of trained immunity operates through a sequential causal scheme connecting receptor-level triggering to long-term epigenetic priming. The cascade initiates when pathogen-associated molecular patterns (e.g., β-glucan or BCG) engage pattern recognition receptors. In human monocytes in vitro, this receptor engagement activates the AKT-mTOR-HIF-1α signaling axis, triggering a marked metabolic shift from oxidative phosphorylation (OXPHOS) to aerobic glycolysis (the Warburg effect) [89]. This glycolytic shift acts as a rate-limiting bottleneck, generating specific metabolites that directly catalyze chromatin remodeling. Rather than being excreted as waste, lactate accumulates and drives histone lactylation (e.g., H3K18la), an emerging modification that sustains open chromatin at pro-inflammatory gene loci [90]. Furthermore, endogenous or exogenous lactate directly promotes chromatin accessibility via histone lactylation, a modification that has been shown to be critical for the trained immune response against bacterial and fungal infections [90]. This glycolytic reprogramming is also observed in sterile inflammation models, where oxidized low-density lipoprotein (oxLDL)-induced training in human monocytes is similarly marked by the concomitant upregulation of glycolysis and oxygen consumption, dependent on enzymes such as PFKFB3 [91]. Beyond glycolysis, glutamine catabolism acts as another crucial metabolic bottleneck in trained immunity. The supplement of glutamine into the TCA cycle results in the accumulation of fumarate. Fumarate functions as a critical signaling molecule by inhibiting KDM5 histone demethylases, thereby sustaining the enrichment of activating histone marks (e.g., H3K4me3) at the promoters of pro-inflammatory cytokines such as TNF-α and IL-6 [92]. Parallel to fumarate, dimethyl itaconate, a derivative of the immune metabolite itaconate, induces long-term trained immunity by remodeling glycolysis and mitochondrial metabolism, enhancing host defense against *Staphylococcus aures* in murine models [93]. Alterations in lipid metabolism further contribute to the regulatory network of trained immunity. Activation of the mevalonate pathway serves as a critical rate-limiting step for the induction of trained immunity. Mevalonate, an upstream intermediate in cholesterol synthesis, facilitates histone modifications and inflammatory responses via the activation of IGF1 receptor and mTOR signaling pathways [94]. Additionally, ceramide metabolism regulates this process; acid ceramidase finely tunes the immune memory of myeloid cells by modulating histone H3K27 acetylation and H3K4 trimethylation [95].

Crucially, this metabolic–epigenetic causal cascade is not restricted to mature peripheral cells but extends to the systemic level. In vivo stimulation of hematopoietic progenitors with β-glucan or BCG induce significant metabolic adaptation, including shifts in glucose metabolism and cholesterol biosynthesis [48]. This metabolic imprinting drives a bias toward myelopoiesis, ensuring the continuous production of metabolically active, epigenetically primed mature myeloid cells and thereby maintaining the trained phenotype for extended periods [48,96].

### 5.2. Metabolic Regulation of T-Cell Memory: Transitions, Maintenance, and Residency

The metabolic trajectory of T cells is not a static state but a dynamic sequence of transitions, where specific metabolites serve as determinants of cell fate. The progression from the quiescent state of naive T cells to the rapid expansion of effector T cells, followed by the development of memory T cells, is regulated by distinct metabolic profiles and mechanisms. Naive T cells remain metabolically quiescent, relying primarily on mitochondrial oxidative phosphorylation for survival. Upon activation via TCR and co-stimulatory signals, T cells undergo rapid metabolic reprogramming, upregulating aerobic glycolysis, glutamine metabolism, and one-carbon metabolism to support biomass accumulation [97,98]. Remodeling of the mitochondrial proteome and enhancement of one-carbon metabolism are highly important for supporting nucleotide synthesis following activation [97,98]. Moreover, in murine cancer models, intracellular levels of L-arginine act as a metabolic bottleneck rather than merely a permissive association, as increased concentrations of L-arginine facilitate a transition from glycolysis to oxidative phosphorylation, thereby enhancing T-cell survival and their anti-tumor efficacy [99]. During the effector-to-memory transition, following antigen clearance, the transition from effector to memory T cells involves a metabolic reversal. Memory T cells diminish their reliance on glycolysis and instead engage in catabolic processes, particularly FAO and mitochondrial oxidative phosphorylation [100]. Mitochondrial dynamics are decisive in this phase and serve as a key rate-limiting determinant; memory T cells maintain tightly fused mitochondrial networks, dependent on Opa1, which tighten electron transport chain complex assembly and enhance spare respiratory capacity [101]. Additionally, downregulation of the mitochondrial pyruvate carrier (MPC) acts as a metabolic gatekeeper that restricts pyruvate oxidation, forcing reliance on fatty acids and glutamine. This metabolic flexibility influences histone acetylation through acetyl-CoA availability and promotes CD8^+^ T-cell differentiation toward a memory phenotype [102].

During homeostatic maintenance metabolites serve as fundamental substrates for epigenetic modifications, directly influencing T-cell lineage choices. The availability of acetyl-CoA, influenced by enzymes such as acetyl-CoA synthetase 2, and ATP-citrate lyase, regulates histone acetylation at loci associated with effector genes and modulates T-cell responsiveness [103,104]. Ketone body metabolism has been identified as a crucial modulator of CD8^+^ T-cell activity. β-hydroxybutyrate serves as an alternative substrate for the tricarboxylic acid (TCA) cycle and enhances histone acetylation, thereby strengthening memory fitness and effector functions [104]. Furthermore, one-carbon metabolism functions as a critical checkpoint in lineage stability. For example, the enzyme MTHFD2 is crucial for sustaining effector functions. Its absence can bias the differentiation of Tregs, underscoring the fine balance of metabolic control in T-cell fate determination [105]. Crucially, this metabolic maintenance is strictly shaped by the local microenvironment for T_RM_ cells. Unlike circulating memory T cells, T_RM_ cells must adapt to tissue-specific lipid availability to sustain long-term residency in barrier tissues.

### 5.3. Metabolic Regulation of B-Cell Memory: GC Dynamics and Fate Decisions

The establishment of long-term humoral immunity is governed by a hierarchical metabolic program spanning GC reactions, fate divergence, and survival niches. The B-cell life cycle encompasses transitions from quiescence to intense proliferation in GCs and finally to high-output protein secretion by LLPCs. Unlike T cells, B cells exhibit distinct metabolic preferences at each differentiation stage. Upon antigen stimulation, naive B cells initiate extensive metabolic reprogramming. Rather than relying solely on glycolysis, activated B cells markedly upregulate oxidative phosphorylation, TCA cycle flux, and nucleotide biosynthesis [106,107,108]. During GC dynamics, B cells proliferate rapidly within a hypoxic microenvironment yet exhibit a highly unconventional metabolic profile. Unlike most rapidly dividing cells, GC B cells display minimal Warburg metabolism and instead rely heavily on fatty acid oxidation for ATP generation [109]. Glucose and amino acids are preferentially allocated to biosynthetic processes rather than energy production. The enzyme MTHFD2 is selectively upregulated to support nucleotide synthesis and redox balance, which are highly important for affinity maturation [110]. Glucose has multiple fates within the B-lymphocyte lineage, including nucleotide production via the pentose phosphate pathway (PPP), lipid synthesis, support of the TCA cycle, and antibody glycosylation [106,111,112,113]. Moreover, asparagine availability controls GC B-cell homeostasis via asparagine synthetase, and its deficiency severely compromises GC reactions [114]. Memory B cells exit the GC reaction and return to a metabolically quiescent state while remaining metabolically poised for rapid reactivation. Autophagy and mitochondrial quality control act as metabolic bottlenecks that are indispensable for long-term survival by preserving cellular homeostasis [115]. In pathological settings such as systemic lupus erythematosus, spleen stromal cell-derived acetylcholine promotes lipid metabolism in B cells through CD36-mediated uptake, driving aberrant autoreactive responses [116]. Terminal differentiation into LLPCs requires adaptation to sustained antibody synthesis and secretion. This process is accompanied by progressive upregulation of oxidative metabolism [117]. The rate-limiting step for LLPCs is the acquisition of nutrients within specific bone marrow survival niches. Unlike GC B cells, plasma cells display high dependence on glucose uptake via GLUT1 [118]. Glucose metabolism serves as the fundamental carbon source for generating the nucleotide sugar donors (e.g., UDP-GlcNAc, UDP-Gal, GDP-Man, GDP-Fuc) required for the biosynthesis of all antibody glycan structures [112]. The glucose-derived carbon enters the pentose phosphate pathway to generate NADPH, which is required to buffer reactive oxygen species (ROS) stress and support antibody production [119]. Collectively, these findings underscore that B-cell immunometabolism not only governs differentiation but also represents a critical determinant of immune homeostasis and disease susceptibility.

### 5.4. The Gut Microbiota Regulates Immune Memory Through Metabolism

**Figure 4:**
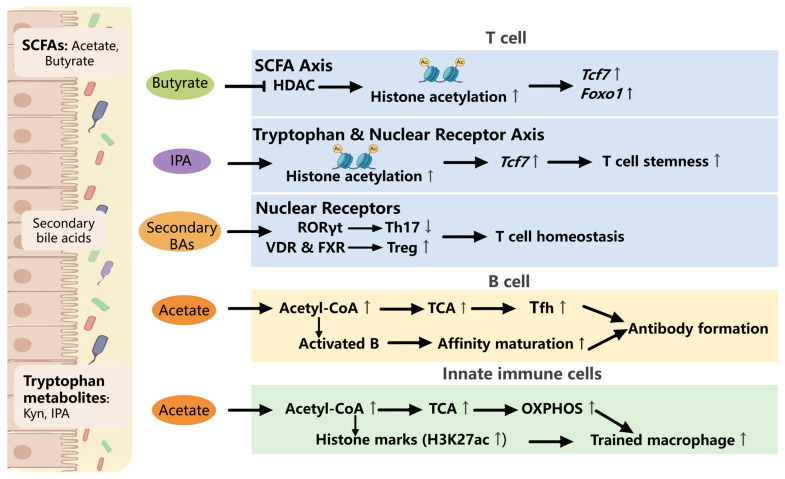
Regulation of gut microbiota-derived metabolites for immune system. Commensal metabolites serve as pivotal modulators integrating bioenergetic flux with chromatin landscape to govern immune cell plasticity. Butyrate-mediated HDAC inhibition promotes locus-specific hyperacetylation (*Tcf7*, *Foxo1*) to drive memory CD8^+^ T-cell differentiation. IPA-driven H3K27ac at the Tcf7 super-enhancer preserves T-cell stemness. Secondary BAs exert divergent nuclear receptor control—inhibiting RORγt while activating VDR/FXR—to orchestrate the Th17–Treg rheostat. Acetate fuels Acetyl-CoA-TCA flux to support Tfh-mediated GC reactions and antibody affinity maturation. Acetate facilitates macrophage trained immunity through integrated metabolic (OXPHOS) and epigenetic (H3K27ac) rewiring. “↑” indicates enhanced response or elevated expression level, “↓” represents reduced response or decreased expression level.

The metabolic regulatory network of immune memory does not operate in isolation but is deeply integrated with the gut microbiota and its metabolite repertoire (Figure 4). Commensal-derived metabolites function as bifunctional regulators, acting as both bioenergetic substrates and instructive signaling and epigenetic cues that collectively shape immune cell metabolism, chromatin architecture, and functional plasticity [120,121]. In murine models of intestinal immunity, short-chain fatty acids (SCFAs), particularly butyrate generated through microbial fermentation of dietary fiber, have been established as metabolic and epigenetic regulators of T-cell memory [122]. Intracellular butyrate functions as a potent inhibitor of histone deacetylases, promoting histone hyperacetylation at immune-relevant loci such as *Tcf7*, *Foxo1*, *Foxp3*, *Ifng*, and *Gzmb*. This remodeling facilitates memory CD8^+^ T-cell differentiation and stabilizes Treg identity [123,124]. In parallel, acetate-derived acetyl-CoA sustains TCA cycle activity, supporting trained immunity in myeloid cells and providing metabolic inputs for antibody biosynthesis in activated B cells in vivo [125,126]. Selected microbiota-derived metabolites also preserve stem-like properties of T cells. Inosine, produced by specific commensal species, can function as an alternative carbon source under glucose-restricted conditions and, in certain contexts, signal through the adenosine A2A receptor–cAMP–protein kinase A axis. While A2AR-mediated signaling is capable of activating CREB-dependent transcriptional programs, direct evidence linking inosine-induced CREB activation to the maintenance of T-cell stemness remains limited [127]. In the context of cancer immunology, microbiota-derived lipid and amino acid catabolites regulate mucosal immune homeostasis through nuclear receptor signaling. Microbiota-derived indole metabolites, including indole-3-acetic acid (IAA) and indole-3-propionic acid (IPA), have been shown in specific mouse tumor contexts to enhance CD8^+^ T-cell-mediated anti-tumor immunity via aryl hydrocarbon receptor (AhR) signaling [128,129]. IAA promotes cytotoxic T-cell responses and supports intestinal barrier integrity, whereas IPA is associated with sustained CD8^+^ T-cell stemness, partly through enhanced H3K27ac and epigenetic remodeling. However, the specific mechanism by which IPA enhances H3K27 acetylation remains to be studied. In contrast, activation of the kynurenine pathway suppresses anti-tumor immunity by depleting tryptophan, inhibiting T-cell proliferation, and promoting Treg differentiation. Although both indole derivatives and kynurenine activate AhR, their divergent immunological outcomes are shaped by ligand-specific signaling, local concentration, and the tissue microenvironment [130,131,132]. Conversely, in systemic vaccine responses, secondary bile acids and tryptophan-derived indole metabolites function as ligands for retinoic acid receptor-related orphan receptor γt (RORγt) and the AhR, respectively [133,134]. These pathways fine-tune Th17–Treg balance, sustain intestinal intraepithelial lymphocyte survival, and support T follicular helper cell (Tfh) differentiation [135,136]. Consequently, gut microbiota-derived signals are supportive of GC formation, affinity maturation, and the establishment of durable B-cell memory [137,138].

Taken together, the establishment of robust immune memory emerges not from isolated biological pathways, but from a tightly intertwined microbiota–metabolism-epigenetics axis. The gut microbiota functions as a continuous upstream environmental sensor, feeding the host’s systemic pool with diverse signaling metabolites (such as SCFAs and indole derivatives) [120,121]. Once internalized by immune cells, these microbial metabolites converge with host-derived intermediates (e.g., lactate, fumarate, and acetyl-CoA) to regulate the epigenetic modification. Specifically, this integrated metabolic pool provides the essential substrates and allosteric regulators for chromatin-modifying enzymes. For instance, while microbial butyrate prevents transcriptional silencing by directly inhibiting histone deacetylases [122,123,124], microbial acetate, and host-derived acetyl-CoA fuel histone acetyltransferases (HATs) to deposit activating marks, such as H3K27ac, at memory-associated enhancers [102,125]. Concurrently, TCA cycle alterations, such as fumarate accumulation, inhibit KDM demethylases to lock in trained immunity signatures (e.g., H3K4me3) [92]. In essence, immunometabolism acts as the physical bridge converting transient microbial and antigenic stimuli into stable, long-lasting epigenetic imprints. Understanding these mechanistic cross-links reveals a translational insight—achieving durable vaccine efficacy requires moving beyond classical antigen–receptor interactions to holistic strategies (Box 2).

Box 2 **Heterogeneity and limitations in immune memory research**

Emerging evidence reveals substantial heterogeneity and limitations in immune memory research that warrant careful consideration. First, species differences, particularly between mouse models and humans, pose challenges for translating mechanistic insights, as variations in memory cell trafficking, niche maintenance, and cytokine dependence may not fully recapitulate human immune dynamics. Second, tissue context significantly shapes memory cell function, with mucosal sites (e.g., lung or gut) and lymphoid organs harboring memory populations exhibiting distinct metabolic, functional, and persistence profiles, yet most current vaccines (e.g., intramuscular delivery) poorly induce robust tissue-resident memory. Third, vaccine platform dependency introduces variability, as mRNA, protein subunit, and viral vector platforms may generate memory cells with divergent durability, recall kinetics, and phenotypic traits, though systematic comparisons across platforms remain limited. Finally, the translatability of immune memory biomarkers, such as surface markers, transcriptional signatures, or metabolic profiles, from animal models to human vaccine efficacy remains uncertain, complicating the prediction of long-term protection in clinical trials. These heterogeneities underscore the need for context-specific evaluations in immune memory research.

## 6. Aging Impacts Immune Memory

**Figure 5:**
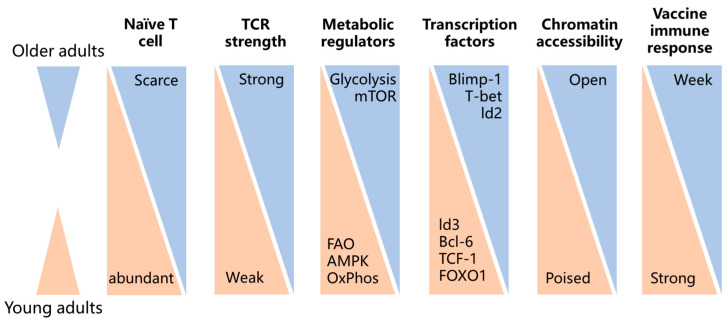
Alterations in signaling, metabolic, and epigenetic signatures between young and old individuals. Significant differences exist in the T-cell status, associated signaling pathways, metabolic patterns, and epigenetic landscapes between young and elderly populations.

Functional changes occur across all human systems with advancing age. In the immune system, aging involves both universal alterations shared among individuals and distinct, person-specific changes [139]. Immune aging is characterized by structural remodeling of immune organs and progressive dysfunction of both innate and adaptive immunity. These changes contribute to impaired vaccine responsiveness and increased susceptibility to infections, age-related diseases, and malignancies [140,141]. The innate immune system shows reduced efficiency in pathogen recognition and clearance, whereas the adaptive immune system undergoes declines in T and B lymphocyte production and function. Collectively, these alterations compromise the ability to mount effective immune responses [142], leading to heightened vulnerability to infections in older adults (Figure 5).

### 6.1. Effect of Aging on Innate Immunity

Immune aging affects virtually all innate immune cell types. Age-related alterations in HSCs within the bone marrow favor differentiation toward myeloid lineages at the expense of lymphoid development [143]. However, neutrophils exhibit impaired superoxide production and reduced phagocytic capacity in aged mice, contributing to immunosuppression [144]. NK cells also undergo functional decline with age, characterized by reduced cytotoxicity and diminished cytokine production [145,146]. Altered cytokine secretion further negatively influences adaptive immune responses [147]. Aging-related changes in dendritic cells in humans include a decrease in plasmacytoid dendritic cells (pDCs) and CD141^+^ myeloid dendritic cell (mDC) subsets [148]. In addition, monocyte-derived dendritic cells (MoDCs) display increased secretion of pro-inflammatory cytokines and reduced production of the anti-inflammatory cytokine IL-10 [149]. Macrophage function is also significantly affected by aging. Upon lipopolysaccharide (LPS) stimulation, aged macrophages produce higher levels of prostaglandin E2, contributing to the altered cytokine milieu observed in older individuals [150]. Collectively, these shifts in cytokine secretion patterns promote the development of chronic low-grade inflammation associated with aging [151], which is closely associated with impaired innate and adaptive immune responses in older adults [152].

### 6.2. Effect of Aging on T Cells

The lineage differentiation dynamics of HSCs in the bone marrow change with age, showing a bias toward myeloid differentiation and resulting in reduced lymphocyte production in older adults [153]. A decline in naïve T-cell numbers and the accumulation of terminally differentiated T cells are two hallmark features of T-cell aging [154]. Together, these changes limit the capacity of the T-cell pool to respond to novel antigens. With advancing age, the TCR repertoire of both CD4^+^ and CD8^+^ T cells undergo progressive contraction, characterized by reduced richness and diversity [155,156]. In particular, the diversity of naïve T cells—critical for shaping effective memory responses—declines significantly [157,158]. Limited homeostatic proliferation of existing T-cell clones, along with diminished thymic output of naïve T cells, further constrains the TCR repertoire in the elderly [158].

In addition, T cells undergo age-related remodeling in both naïve and memory compartments. The stability of the peripheral naïve T-cell pool depends on low-level TCR signaling that supports homeostatic proliferation of recent thymic emigrants, together with cytokine signals mediated by IL-7 and IL-15. With aging, the availability of these key homeostatic cytokine declines, compromising maintenance of the naïve T-cell compartment [159]. This contraction reduces CD4^+^ T-cell activation during immune responses and limits the ability of Tfh to provide essential co-stimulatory and differentiation signals to B cells within the GC [155,160]. Although many studies report minimal age-related decline in total naïve CD4^+^ T-cell numbers [161], naïve CD4^+^ T cells appear more resistant to age-associated loss than naïve CD8^+^ T cells in humans. This difference has been linked to reduced expression of TRIB2 in CD4^+^ T cells [162]. In both humans and mice, TRIB2 deficiency enhances AKT signaling and accelerates IL-7-mediated T-cell proliferation and differentiation during lymphopenia [162]. Within the CD4^+^ T-cell compartment, effector CD4^+^ T cells have been identified among Th1 and Th17 subsets. These cells exhibit pronounced clonal expansion and show increased clonality with advancing age [163,164,165]. In contrast to CD4^+^ T cells, CD8^+^ T cells decline in both absolute numbers and as a proportion of peripheral blood mononuclear cells with age in human [166,167,168]. Aging is also associated with the accumulation of effector memory CD28^−^CD8^+^ T cells and a corresponding decrease in CD28^+^CD8^+^ T cells in the circulation [169,170]. Notably, a GZMK-expressing effector memory CD8^+^ T-cell subset demonstrates age-related expansion and clonal accumulation [171].

Furthermore, senescent T cells undergo metabolic reprogramming characterized by a shift from oxidative phosphorylation to glycolysis [172]. Aging markedly affects both the quality and quantity of mitochondrial DNA (mtDNA). Specifically, mtDNA copy number declines with age in humans, resulting in reduced mitochondrial biogenesis and impaired energy production capacity [173]. Because mitochondria are central to energy-dependent cellular processes, this decline compromises overall cellular function. To compensate, senescent T cells increasingly rely on glycolysis to meet their metabolic demands. Notably, inhibition of p38 MAPK signaling has been shown to enhance mitochondrial biogenesis and autophagy [174], suggesting a potential therapeutic strategy for rejuvenating senescent immune cells and restoring immune function. In addition, chronic infections contribute to immune aging and metabolic reprogramming of T cells by increasing glucose uptake, promoting glycolytic activity, altering lipid raft composition, and disrupting cholesterol homeostasis [175,176].

### 6.3. Effect of Aging on B Cells

Age-related changes in B-cell populations remain incompletely understood and may vary depending on the gating strategies used for analysis. The most commonly applied classification is based on the expression of immunoglobulin (Ig)D and CD27. Some studies reported an age-associated increase in the proportion of naïve B cells (IgD^+^CD27^−^) [177,178,179], whereas others observed no significant change [180] or even a decline [181]. In aging humans or mice, reduced levels of B cell-activating factor (BAFF), which is critical for the survival and maintenance of naïve B cells, limit the replenishment of newly formed B cells [182]. At the same time, memory B cells tend to expand, although they often exhibit a more restricted BCR repertoire. A hallmark of B-cell aging is the accumulation of age-associated B cells, a subset enriched for autoreactive specificities [183]. With advancing age, B-cell functional responsiveness declines. Although the number of memory B cells may be preserved in older individuals following influenza infection, the differentiation capacity of memory B cells into antibody-secreting plasma cells is impaired. This functional defect may be associated with reduced expression of key transcription factors such as Blimp-1 and E47 [184].

### 6.4. Aging Impacts Vaccine Efficacy

Compared with younger individuals, older adults experience more severe infections caused by viruses and bacteria, including influenza, herpes zoster, and pneumococcal disease. However, with advancing age, vaccine-induced immune responses generally decline. This attenuation is characterized by delayed antibody production, lower antibody titers, and impaired T cell-mediated immunity. For example, the protective efficacy of the standard-dose inactivated influenza vaccine reaches 70–90% in children and younger adults but declines to below 50% in individuals aged 65 years and older, substantially reducing its protective benefit [185,186]. The reduced vaccine efficacy observed in older adults may result from age-related alterations in immune cell gene expression, decreased diversity and size of the immune receptor repertoire, and impaired antigen-specific clonal expansion following vaccination [187].

Vaccine-induced neutralizing antibody titers and T-cell responses decline significantly with age. A study of the BNT162b2 COVID-19 vaccine demonstrated a negative correlation between age and both spike (S) protein-specific antibody levels and neutralizing antibody responses [188]. Following the second dose of BNT162b2, neutralizing antibody titers at both the early (day 31) and intermediate (day 105) time points remained significantly lower in older adults (mean age 86 years) compared with younger adults [189]. Age-related differences are also evident in influenza vaccine responses. In a comparative study of young and older adults receiving the trivalent inactivated influenza vaccine (TIV), older participants showed reduced expression of IL-6 and TNF-α and increased expression of IL-10 relative to younger adults [190]. This cytokine shift may dampen the immune response to influenza vaccination in the elderly [191].

Older adults and individuals with underlying comorbidities or immunocompromised conditions are at increased risk of pneumococcal disease and its complications [192]. Pneumococcal polysaccharide vaccines of different valencies generally induce robust serological responses in most older adults. However, immune responsiveness declines progressively with age. This reduced efficacy is associated with downregulation of transcriptional programs involved in T-cell activation and differentiation, NK-cell signaling, and cytokine-related pathways [193].

Reactivation of latent varicella zoster virus (VZV) causes herpes zoster, characterized by painful vesicular rash. The live-attenuated zoster vaccine (ZVL), derived from a high-dose pediatric varicella vaccine, shows decreasing efficacy with advancing age. Its protective efficacy against herpes zoster is 69.8% in individuals aged 50–60 years, 65.5% in those over 60, and 55.4% in those over 70 years [194,195]. In older adults, VZV-specific cytotoxic T lymphocytes display increased markers of senescence and exhaustion compared with younger individuals [196].

### 6.5. Strategies to Enhance Vaccine Immune Responses in Older Adults

Immune aging significantly hinders the generation and durability of immune memory in older adults, making the development of strategies to enhance vaccine-induced immune memory a critical priority. Adjuvanted vaccine (e.g., MF59 and AS01) formulations amplify innate immune activation and antigen availability, promoting stronger memory B- and T-cell responses. Novel platforms like mRNA vaccines further enhance immune memory by improving antigen presentation and sustaining antigen expression, while emerging strategies targeting metabolic and epigenetic pathways aim to extend memory cell longevity. Ultimately, these efforts seek to establish durable, high-quality immune memory capable of providing long-term protection in this vulnerable population.

#### 6.5.1. Increasing Antigen Dosage and Repeated Vaccination

Some vaccines can enhance efficacy by increasing the antigen dose can enhance efficacy by increasing the antigen dose. For example, the high-dose trivalent influenza vaccine formulated for older adults contains four times the standard antigen amount [197]. In clinical trials of the COVID-19 mRNA-1273 vaccine, older adults who received a 100 μg dose exhibited significantly stronger humoral and cellular immune responses than those given a 25 μg dose [198]. In addition to higher antigen doses, repeated vaccination is critical for maintaining humoral immunity in older individuals. Clinical studies have shown that mRNA booster doses significantly enhance immunogenicity against both wild-type and Omicron SARS-CoV-2 variants in older adults [199], while also prolonging the durability of antibody responses [188].

#### 6.5.2. Using Novel Vaccine Adjuvants or New Vaccine Formations

Adjuvants act as immunomodulatory agents that enhance vaccine efficacy in older adults. For example, MF59, a widely used adjuvant in influenza vaccines, facilitates both antigen delivery and immune activation. It promotes chemokine secretion, enhances immune cell recruitment and antigen uptake, improves antigen presentation, and ultimately stimulates adaptive immune responses [200]. MF59 primarily strengthens humoral immunity by increasing anti-hemagglutinin (HA) IgG titers and seroconversion rates in older adults [201]. Studies of RSV vaccines have shown that the AS01B adjuvant enhances immune responses in older individuals by expanding the pool of functionally active antigen-presenting cells (APCs), thereby improving antigen presentation efficiency [202,203]. Overall, adjuvants can partially counteract the effects of immune senescence on vaccine performance. Future research should prioritize the development of novel adjuvants to further improve vaccine immunogenicity in the elderly population.

In contrast to the decreased efficacy of ZVL in older people, a two-dose recombinant zoster vaccine (RZV), which contains glycoprotein E (gE) combined with the AS01B adjuvant, demonstrates superior efficacy compared with ZVL. RZV provides approximately 90% protection in individuals aged 80 years and older, with sustained efficacy exceeding 83% up to eight years after vaccination [204]. RZV also induces stronger antibody responses and greater avidity than ZVL, maintaining elevated gE-specific antibody titers for at least five years [205]. Unlike ZVL, RZV does not show significant age-related decline in gE-specific Th1 responses [206].

Although aging impairs vaccine-induced antibody production and T cell-mediated immunity, booster immunization with COVID-19 vector-based vaccines provides comparable protection across age groups [207]. As for influenza vaccines, substantial progress has been made in the development of nucleic acid-based influenza vaccines in recent years. The enhanced efficacy of mRNA vaccines is attributed to their ability to stimulate innate immunity and promote cytokine production, thereby strengthening both cellular and humoral immune responses [208].

#### 6.5.3. Molecular Target Intervention

As understanding of immune aging advances, several promising therapeutic targets have been identified to mitigate age-related immune decline. AMP-activated protein kinase (AMPK) is a central regulator of cellular energy homeostasis and plays a critical role in immune responses by integrating innate and adaptive immune signaling pathways, thereby influencing immunometabolism and immune cell function [209]. Another key pathway is the mechanistic target of mTOR, a major regulator of cellular lifespan and aging [210]. Rapamycin promotes longevity mainly through inhibition of the mTOR pathway, producing antiproliferative and anti-inflammatory effects and regulating autophagy and apoptosis [211,212]. Phytochemicals such as resveratrol, as well as pharmacological agents including rapamycin and metformin, modulate immune function through these signaling pathways and exhibit anti-aging, antiviral, and immunomodulatory properties. In mice models, resveratrol exerts anti-aging effects primarily by reducing reactive oxygen species (ROS), inhibiting cyclooxygenase (COX) activity, and activating anti-inflammatory pathways in mice, particularly those regulated by Sirtuin-1 (SIRT1) [213]. Metformin activates AMPK, enhances memory T-cell differentiation [214,215], exerts anti-inflammatory effects [216], and reduces ROS production in aging cells [217].

## 7. What Is Ready for Translation, and What Remains Exploratory?

Current research on vaccine-induced immune memory has established several key findings. Well-understood aspects include the mechanisms by which vaccines generate long-term immune memory, such as the formation of memory B cells and long-lived plasma cells in response to antigens. The elucidation of core mechanisms, particularly the metabolic–epigenetic coupling that governs the formation and maintenance of both adaptive immune memory and trained immunity, is under way. Additionally, the role of T follicular helper cells in supporting B-cell responses and the importance of specific adjuvants in enhancing memory formation are also well-documented. However, there are still many areas requiring further investigation. These include the precise molecular and epigenetic mechanisms controlling memory cell differentiation, strategies to optimize vaccine regimens for stronger and longer-lasting memory (e.g., dose, interval, and adjuvant selection), and the development of vaccines that induce robust mucosal immune memory (a current weakness of many intramuscular vaccines). Additionally, researchers are exploring the impact of aging on vaccine efficacy, and the identification of reliable biomarkers for trained immunity. As vaccine research continues to evolve, addressing these gaps will be critical for the development of more effective vaccines.

## 8. Conclusions and Outlook

This review synthesizes current knowledge on the development of immune memory, emphasizing the pivotal roles of metabolic reprogramming and epigenetic regulation in shaping and maintaining both adaptive immune memory and trained immunity. Specially, we pay an attention to the major challenge of aging on immune memory, which substantially reduces vaccine-induced protection in older adults. Future vaccine development may benefit from expanding beyond a traditional focus on adaptive immunity to incorporating insights from trained immunity, immunometabolism, and epigenetic regulation. The relationship between established persistent memory, age-specific adjuvant design, the induction of tissue-resident memory, and biomarkers of trained immunity is the point that requires special attention in future vaccine research. The main strength of this review lies in its comprehensive integration of epigenetic and metabolic regulatory mechanisms across different immune memory paradigms, covering both adaptive immunity (T and B cells) and trained innate immunity, and it also explores the impact of immune aging on immune memory and vaccine efficacy. However, several limitations must be acknowledged. First, as a narrative review, this work is inherently susceptible to selection bias and does not employ the quantitative meta-analytical methods of a systematic review. Second, while we emphasize human cohorts where possible, a substantial portion of the detailed mechanisms discussed (e.g., specific gene knockout models) relies heavily on murine studies, which may not fully recapitulate human immunobiology. Finally, the regulatory mechanisms of immune memory have not yet been well integrated with the design and development of vaccines. By incorporating the emerging knowledge, it may be possible to develop vaccines capable of providing more sustained protection across different age groups.

## Data Availability

No new data were created or analyzed in this study.

## Abbreviations

The following abbreviations are used in this manuscript.

FAO	fatty acid oxidation
GC	germinal center
GDP-Fuc	Guanosine 5′-diphospho-β-L-fucose
GDP-Man	GDP-Mannose
HELLS	Helicase, lymphoid specific
IAA	indole-3-acetic acid
IPA	indole-3-propionic acid
LLPCs	long-lived plasma cells
MMR	Measles, Mumps, and Rubella Vaccine
ncRNAs	non-coding RNAs
OPV	oral poliovirus vaccine
PRC2	Polycomb Repressive Complex 2
TADs	Topologically Associating Domains
TOX	Thymocyte selection-associated high mobility group box
TLR	Toll-like receptor
UDP-Gal	Uridine diphosphate galactose
UDP-GlcNAc	Uridine Diphosphate-*N*-Acetylglucosamine
OXPHOS	oxidative phosphorylation
